# Massive Pulmonary Embolism Complicating Coronavirus Disease 2019 (COVID-19) Pneumonia: A Case Report

**DOI:** 10.1155/2020/8875330

**Published:** 2020-10-27

**Authors:** Shruti Hegde, Gemini Yesodharan, John Tedrow, Alena Goldman

**Affiliations:** ^1^Division of Cardiovascular Medicine, Department of Medicine, St. Elizabeth's Medical Center, Tufts University School of Medicine, USA; ^2^Division of Pulmonary and Critical Care Medicine, Department of Medicine, St. Elizabeth's Medical Center, Tufts University School of Medicine, USA

## Abstract

**Background:**

Patients with severe COVID-19 pneumonia are hypercoagulable and are at risk for acute pulmonary embolism. Timely diagnosis is imperative for their prognosis and recovery. This case describes an otherwise healthy 55-year-old man with respiratory failure requiring mechanical ventilatory support secondary to COVID-19 pneumonia. Massive acute pulmonary embolism with right heart failure complicated his course.

**Case:**

A healthy 55-year-old man presented to our emergency department (ED) with a sore throat, cough, and myalgia. A nasopharyngeal swab was obtained, and he was discharged for home quarantine. His swab turned positive for SARS-CoV-2 infection on real-time reverse transcriptase-polymerase chain reaction assay (RT-PCR) on day 2 of his ED visit. A week later, he represented with worsening shortness of breath, requiring intubation for hypoxic respiratory failure due to COVID-19 pneumonia. Initially, he was easy to oxygenate, had no hemodynamic compromise, and was afebrile. On day 3, he became febrile and developed significant hemodynamic instability requiring maximum vasopressor support and oxygenation difficulty. His ECG revealed sinus tachycardia with S1Q3T3 pattern. On bedside TTE, there was evidence of right heart strain and elevated pulmonary artery systolic pressure of 45 mmHg. All data was indicative of a massive APE as the etiology for his hemodynamic collapse. A decision was made to forgo computed tomography pulmonary angiography (CTPA), given his clinical instability, and systemic thrombolytic therapy was administered. Within the next 12-24 hours, his hemodynamic status significantly improved.

**Conclusions:**

This case highlights the importance of considering massive APE in COVID-19 patients as a cause of the sudden and rapid hemodynamic decline. Furthermore, timely diagnosis can be made to aid in appropriate management with the help of bedside TTE and ECG in cases where CTPA is not feasible secondary to the patient's hemodynamic instability.

## 1. Introduction

After its initial outbreak in Wuhan, China, in December 2019, SARS-CoV-2 has rapidly spread worldwide with 5,405,029 cases as of May 24^th^, 2020 [1, 2].

Our understanding of the etiology of multiorgan failure of severe COVID-19 infection is still incomplete. In critically ill patients with severe COVID-19 pneumonia, there is a correlation between elevated inflammatory markers and markers of hypercoagulability and adverse clinical outcome [3]. High D-dimer levels are commonly associated with venous thromboembolism and acute pulmonary embolism but are difficult to interpret in the setting of severe clinical illness. CTPA is typically done to confirm the diagnosis of APE. However, in cases of rapid clinical deterioration with hemodynamic instability, bedside echocardiogram and electrocardiogram in conjunction with a clinical pretest probability of acute venous thromboembolism can serve as vital diagnostic tools and aids in timely treatment. We describe a case of a previously healthy 55-year old with COVID-19 pneumonia complicated by APE.

## 2. Case Presentation

**Figure 1:**
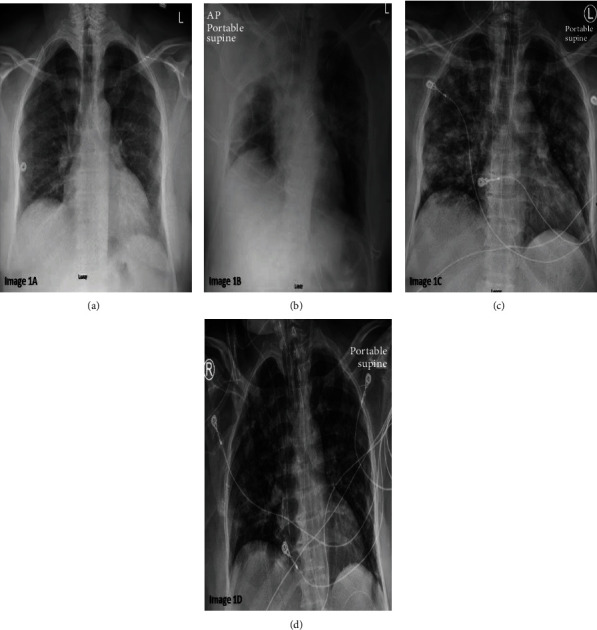
(a) Chest X-ray, PA view on initial presentation to emergency room showing increased interstitial markings bilaterally. (b) Chest X-ray, AP view done on patient's second emergency room visit showing bilateral patchy consolidations and worse in right lung fields. (c) Chest X-ray, PA view on hospital day 3 showing persistent bilateral patchy consolidations improved from hospital day 1. (d) Chest X-ray, PA view on hospital day 7 showing improved bilateral consolidations.

A 55-year-old male without prior medical history presented to the emergency department (ED) with dry cough, sore throat, mouth sores, and myalgias for a week. He was afebrile with stable vitals. Physical examination revealed vesicular lesions on the buccal mucosa and the sublingual area, with posterior pharyngeal erythema, but no edema or midline shift. The lungs were clear. He worked as a taxi driver but denied obvious sick contacts. Chest X-ray (CXR) revealed increased vascular markings, especially at the left base (Figure 1(a)). 12 lead ECG showed sinus rhythm with normal axis, right bundle branch block, and inverted anterior T waves without other changes. Nasal swabs for coronavirus and influenza were obtained, and he was discharged home with instructions for self-quarantine for 14 days. Nasopharyngeal swab turned positive for SARS-CoV-2 on RT-PCR assay in two days.

The patient returned to the ED a week later, with worsening shortness of breath over 24 hours. He was afebrile, with a blood pressure of 143/80 mmHg and a resting heart rate of 88 beats per minute, respiratory rate of 38 per minute, and with oxygen saturation of 88% on 4 liters by nasal cannula. CXR revealed bilateral patchy infiltrates consistent with multifocal pneumonia, right greater than left lung field, and he was intubated with the diagnosis of COVID-19 pneumonia (Figure 1(b)). Laboratory data revealed an unremarkable complete blood count and basic metabolic panel, AST, and ALT of 216 U/L and 159 U/L, respectively. He was stabilized on assist control (AC) ventilation with tidal volume (TV) of 400 mL, positive end expiratory pressure (PEEP) of 12 mmHg, and FiO_2_ of 40%. On these settings, his arterial PaO_2_ was 99%. He was maintained on midazolam drip for sedation. Azithromycin 250 mg once daily and hydroxychloroquine 200 mg Q12 hours were started.

The next day, he became febrile to 102F, and broad-spectrum antibiotics were added to cover for possible bacterial superinfection. Laboratory values were notable for leukocytosis of 14000/*μ*L, creatinine of 1.8 mg/dL, ferritin of 1181 ng/mL, CRP of 163 mg/L, ESR of 90 mm/hr, fibrinogen of 736 mg/dL, and D-dimer of 2.74 *μ*g/mLFEU. His LFTs and ventilator requirements remained stable on hospital day 2.

**Figure 2:**
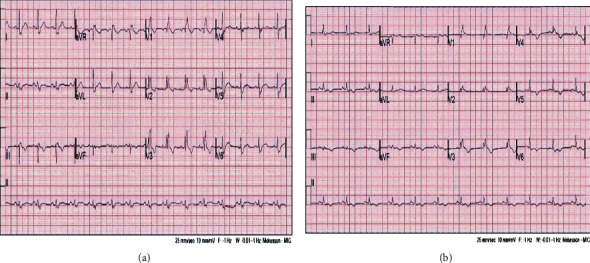
(a) 12 lead EKG on the day of acute pulmonary embolism diagnosis showing sinus rhythm, heart rate of 90 bpm, PR interval of 145 ms, QRS duration of 145 ms, QTc of 420 ms, and QRS axis of 35 degrees. S1Q3T3 RV strain pattern with right bundle branch block (RBBB). (b) 12 lead EKG on day 5 of ICU admission: sinus rhythm, heart rate of 70 bpm, PR interval of 130 ms, QRS duration of 150 ms, QTc of 430 ms, QRS axis of 17 degrees, and RBBB. Diffuse T wave changes.

On hospital day 3, he became progressively hypotensive, tachycardiac, and hypoxic. He quickly escalated to requiring maximum doses of norepinephrine, epinephrine, vasopressin, and phenylephrine. Despite increased ventilator support with FiO_2_ of 100%, PEEP of 14 mmHg, and TV 500 mL, his arterial blood gas (ABG) was notable for a pH of 6.86, pCO_2_ of 84 mmHg, PaO_2_ of 63%, and bicarbonate of 17 mg/dL. His peak inspiratory airway pressure was 25 cmH_2_O and plateau pressure of 24 cmH_2_O. Laboratory data showed worsening leukocytosis to 22,000/*μ*L, creatinine of 3.1 mg/dL, AST of >7000 U/L, ALT 4828 U/L, troponin T < 0.01, CKMB 25.3 ng/mL, NT-pro-BNP of 8244 pg/dL, CRP of 31.6 mg/dL, ferritin 47,646 ng/mL, and D-dimer of >20 *μ*g/mL/FEU (Table 1). Repeat CXR showed persistent bilateral patchy opacities (Figure 1(c)). Bedside 12 lead ECG revealed evidence of RV strain (Figure 2(a)). Bedside echocardiogram revealed severe right ventricular dilation, moderate tricuspid regurgitation, and severely reduced right ventricular function with global hypokinesis ventricle apical sparing, a.k.a. McConnell's sign (94% specificity for the diagnosis of APE) [4] (Movie 1-4). Given hemodynamic instability, a decision was made to proceed directly with systemic thrombolytic therapy in the form of tissue plasminogen activator (alteplase) (TPA) 100 mls at 50 mL/hr. He was then initiated on a heparin infusion as per standard DVT/PE protocol. Over the next day, there was a steady improvement in his hemodynamics. He unfortunately suffered acute oliguric kidney injury and required CVVHD.

By day 5, the patient was weaned off vasopressors. By day 10, he was extubated to nasal cannula. By day 14, at the time of submission, he was discharged to acute rehabilitation facility on room air and apixaban 2.5 mg twice daily given hemodialysis requirement. Repeat CXR and ECG are shown in Figures 1(d) and 2(b), respectively.

## 3. Discussion

We describe a patient with COVID-19 pneumonia complicated by APE, which was diagnosed by bedside tests and successfully treated with thrombolysis.

From the available data from our Chinese colleagues, some of the severe COVID-19 complications correlate with the host inflammatory process and cytokine storm induced by the virus [5, 6]. In our patient, the hypercoagulable state was evident by increased D-dimer and fibrinogen levels, which is not uncommon for the degree of illness. Critically elevated levels of CRP and ESR were observed around the time of APE diagnosis. A heightened inflammatory response resulting in prothrombotic milieu could be one of the possible causes of APE in our patient. Younger patients with a robust immune system may be more susceptible to these complications, and early initiation of immunomodulating therapy is under investigation in this patient subgroup.

Critically ill COVID-19 pneumonia patients fulfill three criteria of Virchow's triad: prolonged immobilization, inflammatory state, and the possibility of endothelial cell damage. A recent study by Obi et al. done on critically ill patients with influenza A H1N1 ARDS, empirical systemic heparin anticoagulation significantly reduced the incidence of pulmonary embolism without increased hemorrhagic complications [7]. A study from Wuhan, China, on 183 consecutive patients with COVID-19 showed that nonsurvivors had markedly elevated levels of D-dimer and fibrin degradation product compared to survivors indicating a higher prothrombotic risk [8]. Empiric therapeutic anticoagulation was used in some severe COVID-19 patients in Wuhan [9]. Initiating empiric anticoagulation, particularly in higher-risk groups of patients, needs more data at this time.

In a recently published study in Lancet [10], retrospective analysis of 25 patients with COVID-19 pneumonia who underwent CTPA due to suspected APE and other clinical concerns found APE in ten patients (40%) of the cohort. Emboli were predominantly located in small branches of the pulmonary artery. The thrombus was partly or entirely resorbed after initiation of systemic anticoagulation therapy for three patients who underwent a follow-up CTPA [10]. In our patient, timely treatment of APE aborted progressive obstructive shock that would have undoubtedly resulted in the patient's demise.

## 4. Conclusion

Our case is one of the few descriptions of a massive APE in a patient with severe SARS-CoV2 pneumonia, promptly diagnosed, and successfully treated with systemic thrombolysis. A bedside echocardiogram and a 12 lead ECG aided in the timely diagnosis of APE as a cause of the sudden hemodynamic collapse and rapid change in oxygenation and ventilation status of our patient. Bedside diagnosis was crucial for both fast therapeutics and avoiding transportation of a critically ill COVID-19 positive patient for an urgent CTPA, thus avoiding cross-contamination and conserving PPE. Further research is required to determine the cause and effect relationship between overactive inflammatory response and risk of development of venous thromboembolic complications in this critically ill patient cohort, as well as to elucidate safety and best anticoagulation choices. Likewise, studies are needed to establish an effect of direct antiviral and immunomodulating therapy on decreasing thromboembolic complications.

## 5. Limitations

The inability to confirm APE with CTPA is a limitation of our case. However, clinical indicators, bedside tests, and clinical response to therapy were sufficient for diagnosis of a massive APE complicating COVID-19 pneumonia.

## Figures and Tables

**Table 1:** Blood work of patient at the time of admission, on the day of event, and on day 8 of admission.

Laboratory measure	Reference range	Day 1	Day 3 (at the time of APE)	Day 5	Day 8
White blood cell (x10^3^/*μ*L)	4.5-11	7.0	21.7	43.1^∗^	37^∗^
Red blood cell (x10^6^/*μ*L)	4.0-5.5	4.99	3.82	3.42	3.78
Hemoglobin (g/dL)	12-17	14.5	11.2	10.4	11.2
Hematocrit (%)	35-50	43.8	37.9	31.5	37.9
Platelet count (x10^3^/*μL*)	150-400	179	131	158	130
ESR (mm/hr)	0-20	90	NA	NA	NA
Sodium (mmol/L)	137-146	137	141	136	135
Potassium (mmol/L)	3.5-5.3	4.0	5.3	4.2	3.9
Chloride (mmol/L)	98-107	99	94	96	98
Bicarbonate (mmol/L)	23-32	26	14	20	24
Blood urea nitrogen (mg/dL)	5-25	10	38	26	42
Creatinine (mg/dL)	0.6-1.4	1.0	1.8	2.4^∗∗^	3.0^∗∗∗^
Ferritin (ng/mL)	30-400	1181	47646	NA	NA
Aspartate amino transferase (AST) (U/L)	15-41	216	>7000	2985	295
Alanine aminotransferase (ALT) (U/L)	14-63	159	4828	3168	975
Troponin T (ng/mL)	≤0.03	<0.01	<0.01	0.77	0.66
NT-pro-B-type natriuretic peptide (NT-pro-BNP) (pg/mL)	0-900	90	8244	3601	2801
Total creatine kinase (U/L)	49-397	133	NA	NA	NA
Creatine kinase MB (CKMB) (ng/mL)	0-6.0	NA	25.3	4.6	2.5
C-reactive protein (CRP) (mg/dL)	<0.5	22.91	31.57	NA	4.93^∗^
C-reactive protein high sensitivity (mg/L)	1.0-3.0	162.7	NA	NA	NA
D-dimer, quantitative (*μ*g/mLFEU)	<0.5	2.74	>20.0	NA	NA
Fibrinogen (mg/dL)	171-459	736	NA	NA	NA
PTT (sec)	23.3-35.9	28	38	75.4^#^	78.2^#^
Arterial blood gas (ABG)	Reference range	VC, FiO_2_ 40%, PEEP 12, TV 400	VC, FiO_2_ 100%, PEEP 14, TV 600	VC, FiO_2_ 40% PEEP 10, TV 400	Spontaneous/CPAP FiO_2_ 40%, PEEP 5
ABG (pH)	7.35-7.45	7.4	7.04	7.41	7.45
ABG partial pressure CO_2_ (mmHg)	35-45	46	63.5	35.1	38.2
ABG partial pressure O_2_ (mmHg)	80-100	215	70.6	189	113
ABG measured O_2_ saturation (%)	94-98	99	90	99.8	99

^∗^On hydrocortisone sodium succinate 50 mg intravenous every 8 hours. ^∗∗^ On continuous veno-venous hemodialysis. ^∗∗∗^ On intermittent hemodialysis. ^#^On therapeutic heparin drip at 15.5 mls/hr (100 units/mL). DVT/PE anticoagulation protocol with heparin 25000 units/250 mL. Bolus dose of 80 units/kg. Initial infusion 18 units/kg/hr. Infusion adjusted to maintain target at PTT of 65-104 seconds. VC: volume control ventilation mode; FiO_2_: fraction of inspired oxygen in percent; PEEP: positive end expiratory pressure in cmH_2_O; TV: tidal volume in milliliter.

## Data Availability

Data are included in the manuscript.

## Additional Points

*Learning Objectives.* (1) Patients with severe coronavirus disease 2019 (COVID-19) pneumonia are hypercoagulable and are at risk for acute pulmonary embolism (APE). (2) Massive acute pulmonary embolism should always be considered in the differential diagnosis for sudden and rapid hemodynamic decline in patients with COVID-19. (3) Critical care bedside echocardiography along with other clinical indicators helps in timely diagnosis and thus treatment of APE in patients with COVID-19 pneumonia, which is lifesaving.

## Supplementary Materials

Supplementary Materials Movie 1A-: transthoracic echocardiography (TEE) parasternal long axis view shows enlarged right ventricle (RV) and a small under filled hyperdynamic left ventricular (LV) cavity. Movie 1B: TTE parasternal short axis view at the level of mitral valve showing enlarged right ventricular (RV) cavity with flattened “D-shaped” interventricular septum (IVS). Movie 1C: TTE apical four-chamber view shows enlarged right ventricle (RV) with severely reduced right ventricular function and global hypokinesis of the right ventricle sparing the apex (McConnell's sign encircled). Also, note the flattening and shift of IVS towards left ventricle (LV). Movie 1D: TTE apical four-chamber view with color Doppler across tricuspid valve (TV) shows moderate tricuspid regurgitation (arrow). Also, seen is the enlarged right ventricle (RV) with severely reduced right ventricular function and McConnell's sign.
